# Methodological challenges and issues of recruiting for mental health and substance use disorders trials in primary care

**DOI:** 10.1186/1940-0640-10-S1-A21

**Published:** 2015-02-20

**Authors:** Anne Marie Henihan, Jan Klimas, Gerard Bury, Thomas O’Toole, Traci Rieckman, Gillian Shorter, Walter Cullen

**Affiliations:** 1 Graduate Entry Medical School, University of Limerick, Limerick, Ireland; 2 School of Medicine and Medical Science, University College Dublin, Coombe Family Practice, Dublin 8, Ireland; 3 Addiction & Urban Health Research Initiative, BC Centre for Excellence in HIV/AIDS, Vancouver, BC, Canada, V6A 1H2; 4 Department of Medicine, Providence VA Medical Center, Providence, RI, 02908, USA; 5 Department of Public Health and Preventive Medicine, Oregon Health & Science University, Portland, OR, 97239-3098, USA; 6 Bamford Centre for Mental Health & MRC, All Ireland Trials Methodology Hub, University of Ulster, Londonderry, Northern Ireland

## Background

Poor recruitment to controlled trials is a frequently reported problem. Challenges related to study design, communication, participants, interventions, outcomes, and clinician workload hinder recruitment, and the effectiveness of interventions used by trialists to increase recruitment rates is unknown.

## Objectives

To explore the methodological challenges and issues in recruiting for mental health and substance use disorder trials in primary care, and to consider how these methodological challenges can be addressed.

## Method

The presentation will recount the authors’ experience of recruiting for cluster randomized trials in primary care. Methodological challenges, such as clarity of instruction, patient characteristics, patient-doctor relationship, effects of intervention on patients and clinic, and personal benefits for clinicians will be described. The authors will consider how these might relate to and be used for peer learning and peer support in primary care research.

## Conclusion

The presentation will conclude with an overview of how lessons learned from past studies may be used to improve recruitment for trials of mental health and substance use disorders in primary care.

